# Changes over time in callus formation caused by intermittently administering PTH in rabbit distraction osteogenesis models

**DOI:** 10.1186/s13018-015-0228-2

**Published:** 2015-06-03

**Authors:** Tetsuya Ohata, Hideto Maruno, Shoichi Ichimura

**Affiliations:** Kyorin University, School of Medicine, 6-20-2 Sinkawa, Mitaka, Tokyo 181-8611 Japan

**Keywords:** Distraction osteogenesis, Teriparatide, Callus formation, Work to failure

## Abstract

**Background:**

Changes over time in the callus during intermittent administration of parathyroid hormone (PTH) were studied in rabbit distraction osteogenesis models.

**Method:**

Models of distraction osteogenesis in Japanese white rabbits were created, and distraction osteogenesis (total length: 10.5 mm) was performed for 2 weeks. Simultaneously with the start of distraction, 30 rabbits received 4 weeks of subcutaneous administration of 30 μg/kg of PTH(1–34), teriparatide, (P-group: *n* = 15) or saline (N-group: *n* = 15) every other day. The tibias of five rabbits were dissected at 6, 8, and 10 weeks after surgery to perform bone mineral density (BMD), peripheral quantitative computed tomography (pQCT), and mechanical testing.

**Results:**

The mean BMD had no significant differences over time at 6, 8, and 10 weeks after surgery between the P-group and the N-group. On pQCT, the P-group had significant increases in total bone cross-sectional area of the callus compared to the N-group at 8 and 10 weeks after surgery. On mechanical testing, the P-group’s absorption energy had not changed at 6 weeks after surgery compared to the N-group, but it had significantly increased at 8 weeks. At 10 weeks after surgery, the N-group’s absorption energy rapidly increased, and the difference between the two groups disappeared.

**Conclusion:**

The intermittent administration of PTH(1–34), teriparatide, for 4 weeks every other day from the start of distraction had the potential to shorten the callus maturation period in the rabbit distraction osteogenesis models.

## Introduction

Distraction osteogenesis has broad-reaching clinical applications in the lengthening of short body height caused by bone diseases [1] and of limbs shortened by various conditions, including comminuted fractures of the long bone shafts, pyogenic osteomyelitis, bone defects after bone tumor resection, and pseudoarthrosis [2]. One of its advantages is that bone grafting is not required even with large bone defects. Conversely, one of the greatest disadvantages clinically is the long period of fitting an external fixation device. This is associated with an increased risk of infection at pin insertion sites for the external fixation device, as well as increased economic and mental burdens on patients because of longer hospitalization.

In order to shorten the treatment period, there are ongoing attempts to combine distraction osteogenesis with other treatment modalities, such as ultrasound [3], extracorporeal shock waves [4], alternating-current electrical stimulation [5], dynamization [6], and administration of various bone metabolism-adjusting drugs and cytokines [7–9]. Bone metabolism-adjusting drugs that could be a candidate for the combination therapy with distraction osteogenesis include bisphosphonates (BPH), which are bone resorption inhibitors, and bone anabolic agents. The former increase bone strength, but the callus that is formed remains without being remodeled, and the mechanical properties of the callus are believed to be inferior to the callus of the normal healing process [10–12]. In contrast, parathyroid hormone (PTH), which is a bone anabolic agent, has had clinical applications in the treatment of osteoporosis, and it has been reported to have significant effects in increasing bone mineral density (BMD) and inhibiting fractures [13]; recently, similar effects were obtained even with intermittent administration given once a week [14]. Experimental studies with distraction osteogenesis models have been reported, and an effect of PTH on callus formation has also been demonstrated in rabbit distraction osteogenesis models [15, 16]. However, no report has examined the detailed changes over time regarding how long the callus-promoting effects of PTH continue.

In this study, changes over time in callus formation were studied during intermittent administration of PTH(1–34), teriparatide, in rabbit distraction osteogenesis models.

## Materials and methods

### PTH preparation

The PTH in this study used is human PTH(1–34), teriparatide acetate (Asahi Kasei Pharma, Tokyo, Japan), which is a sequence of 34 amino acids from the N-terminus. The molecular weight of teriparatide is 4418 Da. A solution of 10 μg/mL of PTH was created using saline as the solvent and then stored frozen at −80 °C in portions of 1-mL containers. The solution was thawed at room temperature immediately prior to administration; the amount of solution corresponding to the weight of each individual rabbit was calculated, and the necessary amount was used for experiments. The same amount of saline was used as a control.

### Animals and test groups

Thirty Japanese white rabbits, weighing 2.2–2.6 kg, were divided into two groups (15 rabbits per group), a 30-μg/kg PTH administration group (P-group), and a saline administration group (N-group) as a control.

### The distraction osteogenesis model

**Fig. 1:**
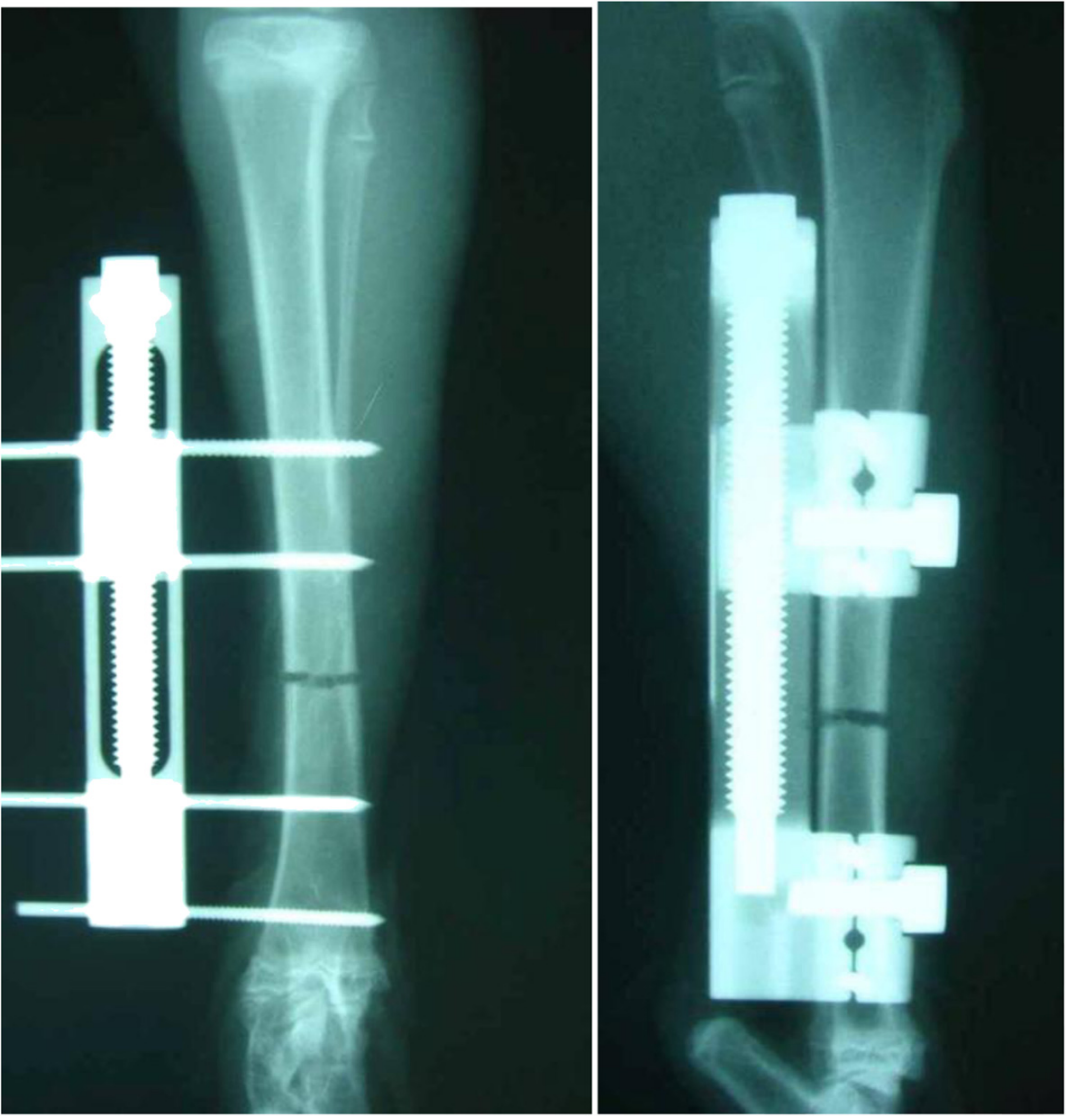
Schematic representation of the callus distraction model. The Orthofix M-100 was fixed to the tibia with four half pins of 2.0 mm. The middle two pins were set at 20 mm apart, and at the center of these, a drill hole osteotomy was performed using a 1.0-mm drill. In order to fit the fragments, an extender is turned in the shortening direction, and the bone fragments were brought into contact with each other

**Fig. 2:**
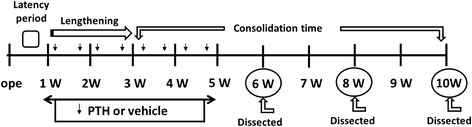
Experimental protocol. The latency period was 1 week, followed by 2 weeks of lengthening and 7 weeks of consolidation time. Either intermittent PTH 30 μg or vehicle was infused locally for 4 weeks from the start of lengthening. The animals were sacrificed at 6, 8, and 10 weeks after operation

The distraction osteogenesis model was based on Maruno’s method [15]. Anesthesia consisted of intramuscular injection of 15 mg/kg of ketamine hydrochloride (Sankyo Seiyaku, Tokyo, Japan), followed by intravenous administration of 30 mg/kg of pentobarbital (Tanabe Seiyaku, Tokyo, Japan). An incision of about 40 mm was made to the medial side of the right hind lower leg, and the periosteum of the medial surface of the tibia was exposed. Without peeling back of the periosteum, two half-pins of 2.0 mm in diameter were punctured so as to straddle the tibiofibular junction with a middle distance of 20 mm between the two pins, and then an external fixator for human short tubular bone (OrthofixM-100; Verona, Italy) was fitted. A drill hole osteotomy was performed at the middle between the two pins, a 10-mm distal site of the tibiofibular junction. During osteotomy, saline was dropping at the osteotomy site to prevent heating. The osteotomy stumps were connected by turning the extender in the shortening direction to make no gap, and then the skin was sutured (Fig. 1). The left hind leg was left untreated. The hind legs were loaded from immediately after surgery, and no limitations were applied to movement. According to the method of Little et al. [10], a 7-day wait after surgery was followed by 14 consecutive days of bone distraction every 12 h, 0.375 mm each time (Fig. 2). The amount of bone distraction was 0.75 mm per day, with a total distraction distance of 10.5 mm, corresponding to about 9.4 % of the total length of the tibia. Simultaneously with the start of distraction, 30 μg/kg of PTH (P-group) or saline (N-group) was administered by subcutaneous injection once every other day for 4 weeks. After the 2 weeks of distraction, rabbits were sacrificed after a 3-, 5-, or 7-week bone-hardening period, at 6, 8, or 10 weeks after surgery, respectively. Then, all rabbits (five rabbits each for two groups at three intervals) were euthanized, and their tibias were dissected after removing the external fixation device. The dissected tibias were wrapped in gauze soaked with saline and stored frozen at −80 °C until testing.

### Measurements

Dissected tibias were naturally thawed at room temperature and subjected to various imaging examinations, and then a three-point bending test was performed.

A. Bone mineral content (BMC) and bone mineral density (BMD)

BMC and BMD of the distraction osteogenesis callus were measured using dual-energy X-ray absorptiometry (DXA) for small animals by the DCS-600EX-R (Aloka, Japan). BMD was measured in 4 mm width up and down from the middle of the distraction osteogenesis callus.

B. Peripheral quantitative computed tomography (pQCT)

A BMD device for small animals, the XCT Research SA+pQCT (Stratec Medizintechnik Gmbh, Pforzheim, Germany), was used to assess the cross-sectional shapes of the distraction osteogenesis callus. Measurements were taken at three sites (the center of the distraction osteogenesis callus and two midpoints between the center and the osteotomy site). Each parameter was measured with a CT width of 0.46 mm, using 690 mg/cm^3^ as a threshold value of typical cortical bone. Measurement parameters were as follows: a) Total-Area (mm^3^); total cross-sectional area of the callus, b) Cortical-Area (mm^3^); cross-sectional area of the cortical bone, measured with a threshold value 690 mg/cm^3^ for typical cortical bone and a threshold value 267 mg/cm^3^ for immature bone cortex, c) Medial-Area (mm^3^); medullary cavity area, which was found by subtracting the cortical bone from the total area, and d) Cortical-Peri (mm); the perimeter of the cortical bone periosteum, e) Cortical-Endo (mm); the perimeter of the cortical bone endosteum, and f) Cortical-Thk (mm); the cortical bone thickness.

C. Mechanical testing (three-point bending test)

A three-point bending test was measured using the GRAPH-2000E (Shimadzu, Japan). The anterior surface of the tibia was faced upward, and the posterior surface was faced downward so that the center of the osteogenesis callus was located at the middle between support points at 20-mm distance. Then, pressure was applied with a crosshead speed by 2 mm/s at the center of the callus from the anterior tibia surface). The measurement parameters were a) absorption energy until callus fracture (work to failure) (N.mm), b) peak force to make callus fracture (peak load) (N), and c) the slope of the linear portion of the load-deformation curve (stiffness) (MPa). The measurement of one of five rabbits in the control group sacrificed at 10 weeks after surgery had failed, and the data was omitted from analysis.

### Statistical analysis

For statistical testing, the IBM SPSS statistics software was used, and significance was set at *p* < 0.05. Numerical values were represented by means ± standard deviation.

### Ethics

This study conformed with Japan’s “Law concerning the protection and control of animals,” “Standard concerning the breeding and protection of laboratory animals,” “Laboratory Animal Guidelines of Kyorin University,” and other relevant guidelines, and it was conducted in a laboratory of Kyorin University’s Department of Orthopedic Surgery.

## Results

### Bone mineral content (BMC) and bone mineral density (BMD)

As shown in Table 1, the mean BMC tended to be higher in the P-group than in the N-group at 6 weeks after surgery, but there were no significant differences between the two groups all through the study period. There were also no significant changes over time in each group. As for the mean BMD, the P-group tended to have higher values at all times, but there were no significant differences between the groups. There were no significant changes over time in each group.

**Table 1:** Bone mineral content and bone mineral density

	6 weeks			8 weeks			10 weeks		
	Control	PTH30	*P*-values	Control	PTH30	*P*-values	Control	PTH30	*P*-values
n	5	5		5	5		5	5	
Bone mineral content (mg)	276 ± 61	475 ± 60	ns	242 ± 58	290 ± 71	ns	305 ± 43	299 ± 66	ns
Bone mineral density (mg/cm^2^)	309 ± 8	379 ± 71	ns	321 ± 56	354 ± 41	ns	253 ± 8	272 ± 37	ns

### Peripheral quantitative computed tomography (pQCT)

a. Total cross-sectional area of the callus (Total-Area)

As shown in Table 2, the Total-Area tended to be greater in the P-group than in the N-group at all times, and the difference was significant at 8 and 10 weeks after surgery (*p* < 0.05). There were no significant changes over time in the Total-Area in each group.

**Table 2:** Measurements with peripheral quantitative computed tomography (pQCT)

	6 weeks			8 weeks			10 weeks		
	Control	PTH30	*P*-values	Control	PTH30	*P*-values	Control	PTH30	*P*-values
n	5	5		5	5		5	5	
Total-Area (mm^3^)	63 ± 8	70 ± 14	ns	64 ± 22	83 ± 13	*P* < 0.05	65 ± 12	82 ± 26	*P* < 0.05
Cortical-Area: 690 mg/cm^3^ (mm^3^)	19 ± 4	22 ± 11	ns	25 ± 7	25 ± 8	ns	21 ± 3	21 ± 5	ns
Cortical-Area: 267 mg/cm^3^ (mm^3^)	60 ± 7	68 ± 10	*P* < 0.05	50 ± 14	54 ± 19	ns	35 ± 7	40 ± 6	ns
Medial-Area (mm^3^)	43 ± 7	50.7 ± 11	ns	29 ± 5	55 ± 8	*P* < 0.05	39 ± 7	54 ± 18	*P* < 0.05
Cortical-Peri (mm)	28 ± 2	30 ± 3	ns	28 ± 5	32 ± 2	ns	28 ± 3	32 ± 5	ns
Cortical-Endo (mm)	23 ± 3	24 ± 3	ns	21 ± 7	29 ± 2	*P* < 0.05	23 ± 4	27 ± 5	*P* < 0.05
Cortical-Thk (mm)	0.7 ± 0.02	0.8 ± 0.4	ns	1.1 ± 0.4	0.9 ± 0.3	ns	0.9 ± 0.2	0.7 ± 0.2	ns

*Total*-*Area* total cross-sectional area of the callus, *Cortical*-*Area* cortical bone cross-sectional area (690 mg/cm^3^: typical cortical bone, 267 mg/cm^3^: including immature bone cortex), *Medial*-*Area* medullary cavity area, *Cortical*-*Peri* perimeter of the cortical bone periosteum, *Cortical*-*Endo* perimeter of the cortical bone endosteum, *Cortical*-*Thk* cortical bone thickness

b. Cortical bone cross-sectional area (Cortical-Area)

In the mean Cortical-Area with a threshold value of 690 mg/cm^3^, there were no significant differences between the two groups. In both groups, the mean value reached a maximum at 8 weeks after surgery, but the changes were not significant over time in each group. On the other hand, the mean Cortical-Area with a threshold value of 267 mg/cm^3^ decreased over time in both groups. The area tended to be greater in the P-group at all times, but there were no significant differences except at 6 weeks after surgery (*p* < 0.05).

c. Medullary cavity area (Medial-Area)

The mean Medial-Area was greater in the P-group than in the N-group at all times, with the differences being significant at 8 and 10 weeks after surgery (*p* < 0.05).

d. Perimeter of the cortical bone periosteum (Cortical-Peri), perimeter of the cortical bone endosteum (Cortical-Endo), and cortical bone thickness (Cortical-Thk)

As for the mean Cortical-Peri, the perimeter tended to be greater in the P-group than in the N-group over time, but there were no significant differences. There were no significant changes over time in each group. In the mean Cortical-Endo, the perimeter tended to be greater in the P-group than in the N-group over time, and there were significant differences at 8 and 10 weeks after surgery (*p* < 0.05). There was no change over time in the N-group, but the perimeter in the P-group tended to be greater at 8 and 10 weeks than 6 weeks after surgery. In the mean Cortical-Thk, there were no significant differences over time between the two groups. The thickness became greater until 8 weeks and then decreased at 10 weeks in both groups, but these changes were not significant.

### Mechanical testing (three-point bending test)

a. Absorption energy until callus fracture (work to failure)

**Fig. 3:**
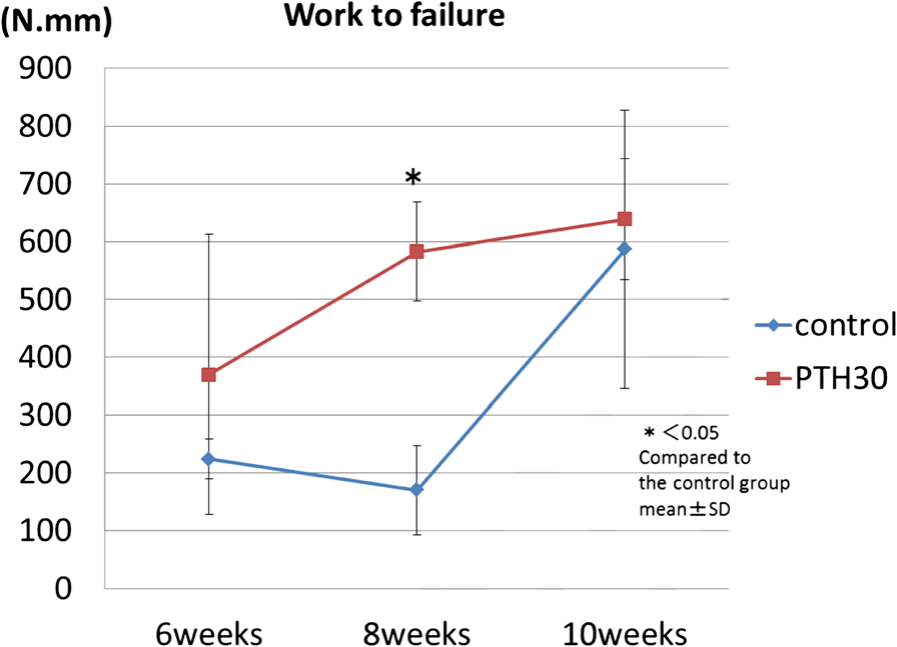
Absorption energy until callus fracture (Work to failure). The mean work to failure increases significantly in the PTH30 μg group compared with the control group at 8 weeks after operation. The change has almost disappeared at 10 weeks after operation

As shown in Table 3, the mean value of work to failure increased significantly in the N-group at 10 weeks after surgery (*p* < 0.05) compared to 6 and 8 weeks. In the P-group, it increased significantly at 8 weeks and substantially reached a plateau at 10 weeks after surgery. The energy tended to increase more rapidly in the P-group than in the N-group at 6 weeks, and it reached to be significant at 8 weeks after surgery. At 10 weeks, however, the N-group also rapidly increased, and there was no longer a significant difference between the two groups (Fig. 3).

**Table 3:** Structural properties by three-point bending analysis

	6 weeks			8 weeks			10 weeks		
	Control	PTH30	*P*-values	Control	PTH30	*P*-values	Control	PTH30	*P*-values
n	5	5		5	5		4^a^	5	
Work to failure (N.mm)	224 ± 35	370 ± 243	ns	170 ± 77	583 ± 86^b^	*P* < 0.05	587 ± 241^c^	639 ± 105^b^	ns
Peak load (N)	326 ± 40	474 ± 177	ns	304 ± 72	436 ± 156	ns	443 ± 204	339 ± 50	ns
Stiffness (MPa)	337 ± 90	454 ± 225	ns	454 ± 39	427 ± 72	ns	389 ± 66	338 ± 148	ns

*Work to failure* absorption energy until callus fracture, *peak load* peak force to make callus fracture, *stiffness* the slope of the linear portion of the load-deformation curve

^a^The data from one of five rabbits were omitted due to experimental failure

^b^ *P* < 0.05, compared to 6 weeks in PTH30 group

^c^ *P* < 0.05, compared to 6 and 8 weeks in control group

b. Peak force to make callus fracture (peak load)

There were no significant differences between the two groups over time. And there were no significant changes over time in each group.

c. Slope of the linear portion of the load-deformation curve (Stiffness)

Stiffness tended to increase at 6 and 8 weeks after surgery in the N-group, while in the P-group, stiffness showed the greatest value at 6 weeks after surgery and gradually decreased at 8 and 10 weeks after surgery. There were no significant differences over time in the two groups.

## Discussion

The greatest advantage of distraction osteogenesis for shorter limbs is that it does not need a donor bone such as vascularized bone grafts. However, distraction osteogenesis requires a bone-hardening period for maturation of the callus, and it usually requires two to three times longer than the distraction period. Shortening the callus maturation period is one of the greatest problems, which should be resolved in distraction osteogenesis, but there has been no useful combination therapy in clinical practice.

PTH is a polypeptide hormone composed of 84 amino acid residues, but its biological activity resides in 1–34 of the N-terminus region. This human PTH(1–34), teriparatide, is known to promote both bone formation and bone resorption when administered intermittently in rats. Since the increase in bone formation is greater than that in bone resorption, bone mass increases as a result [17].

Effects of intermittent PTH(1–34) administration on cancellous bone are thought to involve improving the bone microarchitecture and enhancing bone strength, such as by increasing the connectivity of the trabecula and cancellous bone mass, and shifting the trabecular structure from rod-like to plate-like structure [18]. As a result, the lumbar spine BMD increases and new vertebral fractures are diminished clinically [13]. For cortical bone, PTH(1–34) has been thought to promote the cortical porosity and reduce the cortical bone width and cortical bone strength. However, the cortical bone width in the ilium did increase significantly, and there was no significant change in porosity [18–20]. In the study using monkey tibias and femurs, bone strength was increased by greater thickness of the cortical bone [21]. PTH(1–34) also increased cortical thickness and cortical porosity in tibia and radius clinically, however, bone strength was not deteriorated and similar as zoledronic acid treatment [22]. Recently, treatment of PTH(1–34) for 2 years has shown to reduce non-vertebral fragile fracture [23].

There have also been numerous reports on basic research for fracture healing with intermittent PTH administration. In rat femur fracture models, PTH increased the BMC, the ultimate load, the callus mass, the callus area, and callus strength [24–29]. And PTH had an effect in promoting temporal and volume-dependent cortical ossification at fracture sites [24]. PTH is thought to promote fracture healing. Furthermore, expansion of the periosteal diameter increases cortical bone strength, and this phenomenon is called cortical drift, and PTH promoted cortical drift, which effects on the cortical bone were caused by the periosteal and endocortical membrane reactions [21].

On the other hand, there were few reports about the effects of intermittently administering PTH(1–34) on bone distraction osteogenesis [15, 16, 30–32]. Seebach et al. used rat femur distraction models by administering PTH daily, and with a 20-day bone-hardening period, the callus volume and BMD increased 58 and 24 %, respectively, and the stiffness and ultimate load increased 50 %. With the 40-day bone-hardening period, bone mass also increased similarly, but they reported that the 20-day bone-hardening period showed a greater effect [30]. However, cortical bone remodeling in rats, which are a species of rodent and therefore have no Haversian system, is believed to be higher response to PTH than in humans. Aleksyniene et al. [16, 32] used a distraction osteogenesis model with rabbit tibias, an animal with the same remodeling as humans. There was an increase in BMD and bone strength in a group of daily PTH administration for a 10-day distraction period followed by a 20-day bone-hardening period. And a group of daily PTH administration for only a 20-day bone-hardening period showed the same results as the former group. Maruno et al. [15] performed intermittent administration of 10 or 30 μg/kg of PTH every other day in rabbit distraction osteogenesis models. They administered PTH for 4 weeks after the start of a 14-day distraction with a 5-week bone-hardening period. They reported that the PTH 30 μg/kg group had increased cortical bone formation and callus width, and the callus strength was significantly greater than PTH 10 μg/kg and control groups. However, the duration of this positive effect by PTH was unknown. In the present study, PTH was administered every other day, and the bone-hardening period was changed to 1.5-fold (6 weeks after surgery), 2.5-fold (8 weeks after surgery), or 3.5-fold (10 weeks after surgery) after a 14-day distraction in order to investigate the changes in the course of distraction callus formation.

**Fig. 4:**
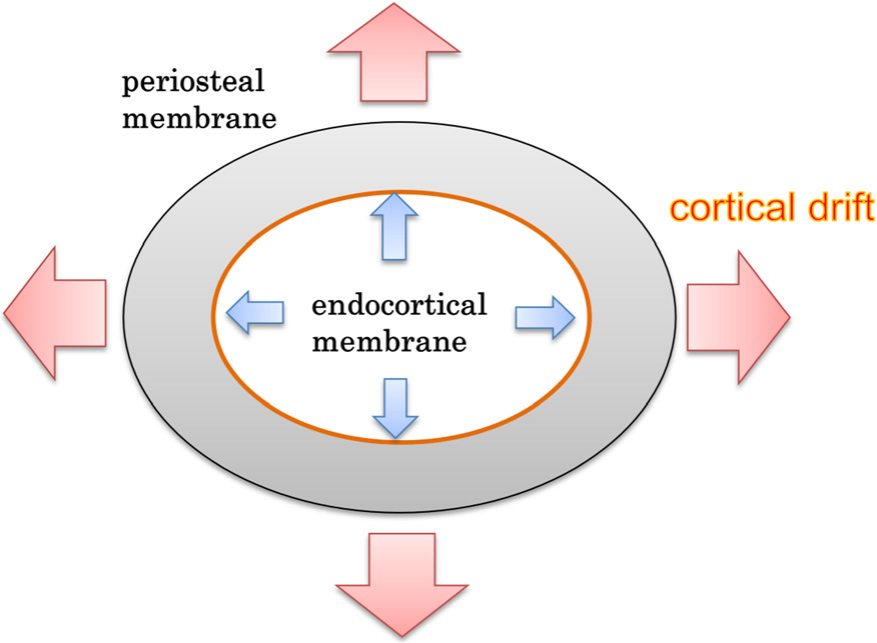
Cortical drift. Intermittently administering PTH affected the periosteal and endocortical membrane, with the callus expanding outward, and the overall volume becoming greater

BMD did not change over time in this study. Bone strength, however, did increase significantly in the P-group at 8 weeks after surgery compared to the N-group, and there was no longer a difference at 10 weeks. This result seemed to mean that PTH’s effect of enhancing bone strength occurred in a bone-hardening period that was 3–5 weeks after distraction (8–10 weeks after surgery) in this model. Considering this results in pQCT, the mean total cross-sectional area of the distraction callus had no significant change over time in each group, but the P-group values were greater than the N-group values at all times, and the P-group had a significantly greater cross-sectional area at 8 and 10 weeks after surgery. For mean cortical bone cross-sectional area with a threshold value of 267 mg/cm^3^, which included immature bone, the P-group increased significantly compared to the N-group at 6 weeks after surgery, and the values also tended to be greater at a threshold value of 690 mg/cm^3^ for typical bone cortex, suggesting that the effects of administering PTH were already observed at 6 weeks after surgery. For medullary cavity area, the fact that the P-group had significantly increased compared to the N-group at 8 and 10 weeks after surgery implied that PTH accelerated the resorption of the immature bone inside the medullary cavity and promoted the remodeling of the distraction callus. This results resembled the appearance of cortical drift as in normal bone changes, and the distraction callus extended outward and the overall bone volume was increased (Fig. 4).

In terms of the changes over time in callus formation by intermittently administering PTH in the present study, an acceleration of remodeling had already appeared for the 21-day bone-hardening period after distraction (at 6 weeks after surgery). An increase in the periosteal diameter associated with enhancing of the periosteal ossification of the cortical bone for a 35-day bone-hardening period (at 8 weeks) was observed, and bone strength was accelerated as a result of enhanced bone remodeling. For 49 days of the bone-hardening period (at 10 weeks), the bone strength of the N-group was rapidly caught up to that of the PTH group, and there was no difference between the two groups. Intermittently administering PTH has the potential to accelerate the bone remodeling and reduce the callus maturation period in distraction osteogenesis.

## Conclusions

Intermittently administering PTH for 4 weeks from the start of distraction in rabbit distraction osteogenesis models accelerated remodeling of the callus from the early stage of from 21 to 49 days after distraction compared to the control group and reduced the callus maturation period by about 2 weeks, therefore demonstrating its usefulness for distraction osteogenesis.

## Abbreviations

PTH: parathyroid hormone
BMD: bone mineral density
pQCT: peripheral quantitative computed tomography
